# Supplementary material for: HIVprotI: an integrated web based platform for prediction and design of HIV proteins inhibitors
Source: J Cheminform. 2018 Mar 9;10:12. doi: 10.1186/s13321-018-0266-y (PMC5845081; doi:10.1186/s13321-018-0266-y)
Supplement: Supplementary file 2 — Additional file 2. Source code of HIVProtI web server. [file 13321_2018_266_MOESM2_ESM.zip › HIVprotI_Source-code/hivprotI/weka-3-6-6/doc/weka/gui/boundaryvisualizer/RemoteBoundaryVisualizerSubTask.html]

RemoteBoundaryVisualizerSubTask


---


|  |  |  |
| --- | --- | --- |
| **Overview** \| **Package** \| **Class** \| **Tree** \| **Deprecated** \| **Index** \| **Help** \| **Weka's home** | |  |
| **PREV CLASS**   **NEXT CLASS** | **FRAMES**    **NO FRAMES**     **All Classes** |  |
| SUMMARY: NESTED \| FIELD \| CONSTR \| METHOD | DETAIL: FIELD \| CONSTR \| METHOD |  |


---


## weka.gui.boundaryvisualizer Class RemoteBoundaryVisualizerSubTask

```
java.lang.Object
  weka.gui.boundaryvisualizer.RemoteBoundaryVisualizerSubTask
```

**All Implemented Interfaces:**: java.io.Serializable, Task

---

``` public class RemoteBoundaryVisualizerSubTask extends java.lang.Object implements Task ```

Class that encapsulates a sub task for distributed boundary
visualization. Produces probability distributions for each pixel
in one row of the visualization.

**Since:**
:   1.0

**Version:**
:   $Revision: 7059 $

**Author:**
:   Mark Hall

**See Also:**: `Task`, Serialized Form

---

**Constructor Summary**

`RemoteBoundaryVisualizerSubTask()`


| **Method Summary** | |
| --- | --- |
| `void` | `execute()`             Perform the sub task |
| `TaskStatusInfo` | `getTaskStatus()`             Return status information for this sub task |
| `void` | `setClassifier(Classifier dc)`             Set the classifier to use |
| `void` | `setDataGenerator(DataGenerator dg)`             Set the density estimator to use |
| `void` | `setGeneratorSamplesBase(double ksb)`             Set the base for computing the number of samples to obtain from each generator. |
| `void` | `setInstances(Instances i)`             Set the training data |
| `void` | `setMinMaxX(double minx, double maxx)`             Set the minimum and maximum values of the x axis fixed dimension |
| `void` | `setMinMaxY(double miny, double maxy)`             Set the minimum and maximum values of the y axis fixed dimension |
| `void` | `setNumSamplesPerRegion(int num)`             Set the number of points to uniformly sample from a region (fixed dimensions). |
| `void` | `setPanelHeight(int ph)`             Set the height of the visualization |
| `void` | `setPanelWidth(int pw)`             Set the width of the visualization |
| `void` | `setPixHeight(double ph)`             Set the height of a pixel |
| `void` | `setPixWidth(double pw)`             Set the width of a pixel |
| `void` | `setRowNumber(int rn)`             Set the row number for this sub task |
| `void` | `setXAttribute(int xatt)`             Set the x axis fixed dimension |
| `void` | `setYAttribute(int yatt)`             Set the y axis fixed dimension |

**Methods inherited from class java.lang.Object**

`equals, getClass, hashCode, notify, notifyAll, toString, wait, wait, wait`

**Constructor Detail**

### RemoteBoundaryVisualizerSubTask

```
public RemoteBoundaryVisualizerSubTask()
```


**Method Detail**

### setRowNumber

```
public void setRowNumber(int rn)
```

:   Set the row number for this sub task

    :   **Parameters:**: `rn` - the row number

---


### setPanelWidth

```
public void setPanelWidth(int pw)
```

:   Set the width of the visualization

    :   **Parameters:**: `pw` - the width

---


### setPanelHeight

```
public void setPanelHeight(int ph)
```

:   Set the height of the visualization

    :   **Parameters:**: `ph` - the height

---


### setPixHeight

```
public void setPixHeight(double ph)
```

:   Set the height of a pixel

    :   **Parameters:**: `ph` - the height of a pixel

---


### setPixWidth

```
public void setPixWidth(double pw)
```

:   Set the width of a pixel

    :   **Parameters:**: `pw` - the width of a pixel

---


### setClassifier

```
public void setClassifier(Classifier dc)
```

:   Set the classifier to use

    :   **Parameters:**: `dc` - the classifier

---


### setDataGenerator

```
public void setDataGenerator(DataGenerator dg)
```

:   Set the density estimator to use

    :   **Parameters:**: `dg` - the density estimator

---


### setInstances

```
public void setInstances(Instances i)
```

:   Set the training data

    :   **Parameters:**: `i` - the training data

---


### setMinMaxX

```
public void setMinMaxX(double minx,
                       double maxx)
```

:   Set the minimum and maximum values of the x axis fixed dimension

    :   **Parameters:**: `minx` - a `double` value: `maxx` - a `double` value

---


### setMinMaxY

```
public void setMinMaxY(double miny,
                       double maxy)
```

:   Set the minimum and maximum values of the y axis fixed dimension

    :   **Parameters:**: `miny` - a `double` value: `maxy` - a `double` value

---


### setXAttribute

```
public void setXAttribute(int xatt)
```

:   Set the x axis fixed dimension

    :   **Parameters:**: `xatt` - an `int` value

---


### setYAttribute

```
public void setYAttribute(int yatt)
```

:   Set the y axis fixed dimension

    :   **Parameters:**: `yatt` - an `int` value

---


### setNumSamplesPerRegion

```
public void setNumSamplesPerRegion(int num)
```

:   Set the number of points to uniformly sample from a region (fixed
    dimensions).

    :   **Parameters:**: `num` - an `int` value

---


### setGeneratorSamplesBase

```
public void setGeneratorSamplesBase(double ksb)
```

:   Set the base for computing the number of samples to obtain from each
    generator. number of samples = base ^ (# non fixed dimensions)

    :   **Parameters:**: `ksb` - a `double` value

---


### execute

```
public void execute()
```

:   Perform the sub task

    :   **Specified by:**: `execute` in interface `Task`

---


### getTaskStatus

```
public TaskStatusInfo getTaskStatus()
```

:   Return status information for this sub task

    :   **Specified by:**: `getTaskStatus` in interface `Task`
    :   **Returns:**: a `TaskStatusInfo` value


---


|  |  |  |
| --- | --- | --- |
| **Overview** \| **Package** \| **Class** \| **Tree** \| **Deprecated** \| **Index** \| **Help** \| **Weka's home** | |  |
| **PREV CLASS**   **NEXT CLASS** | **FRAMES**    **NO FRAMES**     **All Classes** |  |
| SUMMARY: NESTED \| FIELD \| CONSTR \| METHOD | DETAIL: FIELD \| CONSTR \| METHOD |  |


---
